# Pigment removal from reverse-printed laminated flexible films by solvent-targeted recovery and precipitation

**DOI:** 10.1126/sciadv.adt5841

**Published:** 2025-03-14

**Authors:** Tianwei Yan, Charles Granger, Kevin L. Sánchez-Rivera, Panzheng Zhou, Steven Grey, Kevin Nelson, Fei Long, Ezra Bar-Ziv, Reid C. Van Lehn, Styliani Avraamidou, George W. Huber

**Affiliations:** ^1^ Department of Chemical and Biological Engineering, University of Wisconsin-Madison, Madison, WI 53706, USA.; ^2^ Amcor, Neenah Innovation Center, Neenah, WI 54956, USA.; ^3^ Department of Mechanical Engineering, Michigan Technological University, Houghton, MI 49931, USA.; ^4^ Department of Chemistry, University of Wisconsin-Madison, Madison, WI 53706, USA.

## Abstract

The solvent-targeted recovery and precipitation (STRAP) process separates and recovers the constituent resins in multilayer plastic packaging films by selective polymer dissolution. In this work, the cause of coloring in the STRAP-recycled polyethylene (PE) resins from postindustrial printed films was identified as decomposed diarylide pigments. Two different approaches are needed to completely remove the dissolved colorants during the STRAP process including (i) adding an activated carbon (AC) adsorbent to the solvent after polymer dissolution and (ii) proper mechanical filtration of the polymer-solvent cake to remove as much solvent from the cake as possible. Colorless recycled PE can be produced by a combination of the proposed approaches (choosing the proper solvent, adding an AC adsorbent, and doing proper mechanical filtration) with minimal accumulation of colorants in the recycled STRAP solvents. This study demonstrated that high-quality STRAP low-density PE can be obtained from printed plastic films, enhancing the potential circularity of these packaging materials.

## Teaser

Analysis revealed the color source of polyethylene recovered from multilayer films and inspired techniques to reduce the color.

## INTRODUCTION

Over the last ~70 years, plastic materials have become indispensable to modern society beginning with their large-scale production after World War II (*1*). Their durability, low cost, and versatility have driven an exponential growth of plastics production (*1*–*3*). However, this growth has led to end-of-life issues, such as waste accumulation and environmental plastics contamination.

Flexible plastic packaging is particularly challenging to recycle as it typically includes several layers of distinct polymers for their moisture and oxygen barrier properties, sealability, and mechanical strength (*4*, *5*). Tie layers, adhesives, additives, and inks add further complexity to the composition, resulting in incompatibility with traditional recycling technologies (*5*). Ineffective separation and purification of different polymers result in downgraded recyclates with deteriorated properties.

Virgin plastics are usually colorless plastic materials while inks are typically added to most plastics to improve the aesthetic demands for products (*6*). After mechanical recycling, colorants often cause recycled plastics to appear gray or black (*7*), making them distinguishable from colorless virgin plastics. A recent report evaluated mechanically recycled low-density polyethylene (LDPE) printed flexible packaging with pigments and ink binders (*8*). The results indicated that the presence of ink introduced yellowish or brownish tones to the recycled films as well as reduced transparency and pigment aggregates, e.g., “black specks.”

Color removal from recycled polymers is difficult because the high color strength of colorant species and high color sensitivity of human eyes lead to recognizable color in the recycled plastic materials even with minimal retention of colorants. The retained colors can adversely affect customer perception of the recycled plastic products (*9*). For example, yellow color is commonly associated with age and degradation (*10*–*12*). The adverse impact of colors in recycled resins is reflected in the price difference between colored and colorless postconsumer plastics (*8*, *13*–*17*). Taking high-density polyethylene (HDPE) as an example, the price of mixed color HDPE bales is one-half to one-third of colorless baled HDPE (*8*, *13*–*17*). Therefore, effective removal of colors from recycled polymers is critical for improving the product value of recyclates.

Understanding colorants is crucial for effective color removal. Colorants are diverse in chemical compositions and physical properties and are classified as pigments or dyes. Pigments are designed to reside in discrete particles while dyes are designed to be soluble in solvents (*18*). For plastic packaging printing, organic pigment–based inks are most common because dyes tend to bleed and leach with incompatible plastics (*6*). Pigments require binders and additives in the inks to disperse properly on printed areas. A myriad of chemical compositions and structural variants bring distinct physical and chemical properties. Making it challenging to remove all pigments with one single method. Common colorants and their chemical families are listed in table S1.

The undesired color and poor appearance of recycled plastics have troubled plastic recyclers since the 1980s (*19*–*23*). Deinking pretreatments have been developed to remove inks from a plastic’s surface (*24*). However, when ink is printed on an inner layer of flexible packaging (i.e., reverse-printed laminated films), the effectiveness and rate of deinking are compromised (*25*). Consequently, recycling technologies are emerging to create higher-quality recycled plastics. Dissolution-based recycling has gained interest as a complementary method to traditional mechanical recycling. This approach can extract valuable polymer components from multilayer films and remove additives and impurities with distinct solubilities (e.g., pigments) (*24*, *26*, *27*).

**Fig. 1.**
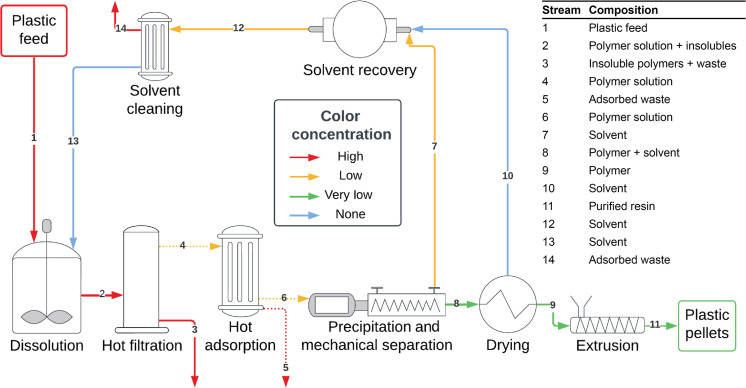
Conceptual illustration of the STRAP process. A multilayer plastic waste is fed into the process (stream 1) and a single purified polymer is recovered (stream 11) with nearly all color removed. “Hot adsorption” is an optional step, indicated by dashed streams. For more information on the STRAP process, see (*5*).

Solvent-targeted recovery and precipitation (STRAP) is a dissolution-based process that recycles multilayer films by using a series of solvents to selectively recover the constituent resins of the material guided by thermodynamic calculations of polymer solubility (*5*, *25*, *28*–*30*). The STRAP process has been successful in recovering polyethylene (PE), polypropylene (PP), ethylene vinyl alcohol (EVOH), and polyethylene terephthalate (PET) from multilayer films with high purity, efficiency, and reduced color (*25*, *28*). The STRAP process units (Fig. 1) include the following:

1) Dissolution: shredded plastics are mixed with a hot solvent to dissolve the targeted layer.

2) Hot filtration: hot dissolved plastics are separated from solid residue.

3) Precipitation: the polymer solution is cooled to precipitate the plastic (antisolvents are not used in STRAP to minimize costs and environmental impact) (*31*).

4) Mechanical separation: solvent is mechanically separated from precipitated polymer.

5) Drying: solvent retained in the polymer is evaporated.

6) Extrusion: the polymer is extruded and pelletized.

7) Solvent recovery: as much solvent as possible is recovered to be recycled.

8) Solvent cleaning: solvent is purified, ideally with adsorbents, to avoid contaminant buildup.

Colorants that pass through the hot filtration step could be trapped in the purified polymer during the precipitation step. Colorants in general have a low volatility and cannot be removed during the drying step shown in Fig. 1; thus, any soluble colorants would likely deposit onto the recovered polymer.

In two previous cases, STRAP removed white, yellow, and black colorants from PET and blue colorants from PP via selective dissolution of the inks (*25*, *28*). In these instances, colorants were removed from the color-rich final products in a secondary wash step. This extra wash step adds additional cost, emissions, and energy consumption (*17*). Therefore, we aim to optimize the color removal approach.

**Fig. 2.**
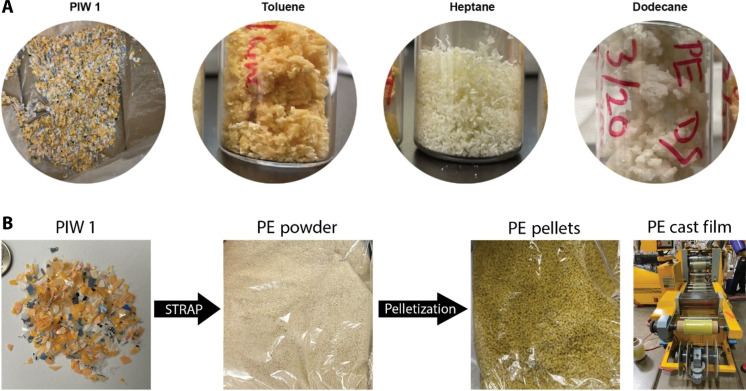
Recovered PE from a reverse-printed laminated multilayer film via STRAP. (**A**) The coloring issue was mitigated after solvent optimization (*25*). (**B**) The cast film made from STRAP resins was still noticeably yellow (*32*).

We also previously attempted to isolate colorless postindustrial waste (PIW 1) PE from a multilayer reverse-printed laminated polyester film using a single solvent STRAP system (*25*). The resulting PE was then manufactured into a cast film and characterized (*32*). Although we removed a large fraction of the colorants (Fig. 2A), the film still had a light but noticeable yellow color (Fig. 2B) (*25*, *32*). Ideally, colorless recycled plastic should be produced using a single solvent to maximize financial and environmental benefits, given the high impact of organic solvents (*17*, *25*, *32*).

Other dissolution-based methods have observed similar issues. For example, Creasolv’s Indonesia plant produces yellow-green recycled PE (rPE) from waste sachets (*19*, *33*), likely caused by remaining mixed pigments and other impurities. The yellowing of plastics, however, is common and not limited to dissolution-based recycling. Causes of yellowing can include oxidation of phenolic antioxidant (*34*), polymer degradation (*35*–*37*), pigment retention (*25*, *28*), catalyst choice (*38*), and formation of chiral supramolecular structure via irradiation (*12*). Degradation of the bulk polymer, adhesives, or ink binders in printed films may produce yellow colorants in recycled plastic (*1*, *12*, *34*, *36*, *37*).

In this work, we aim to identify the composition of remaining colorants and demonstrate a method for the nearly complete removal of color from reverse-printed laminated multilayer flexible films made of PE, EVOH, and PET, with polyurethane (PU) inks. The challenge is to identify the color source in the recovered resins and understand colorant solubility and accumulation during the STRAP process. We then show how the processing of the reverse-printed laminated film can produce colorless polymer resins.

## RESULTS

### Color source in rPE from STRAP

To determine the exact cause of rPE coloring after the STRAP process, we first conducted experiments with various ink-free films to confirm that other components do not cause yellowing during STRAP (*5*). Therefore, the possible coloring components in reverse-printed laminated films are PU adhesives and inks. PU adhesives are used to bind together different layers in a multilayer film. Inks consist of solvents, colorants, and additives for printing, durability, and other aesthetic needs (*8*, *39*). Pigment-based inks also require the presence of binders to properly disperse pigments on the printed area. PU and nitrocellulose (NC) are the most common ink binders in multilayer films (*8*). Any of these components can potentially cause discoloration when recycled (*8*, *12*, *35*, *36*).

**Fig. 3.**
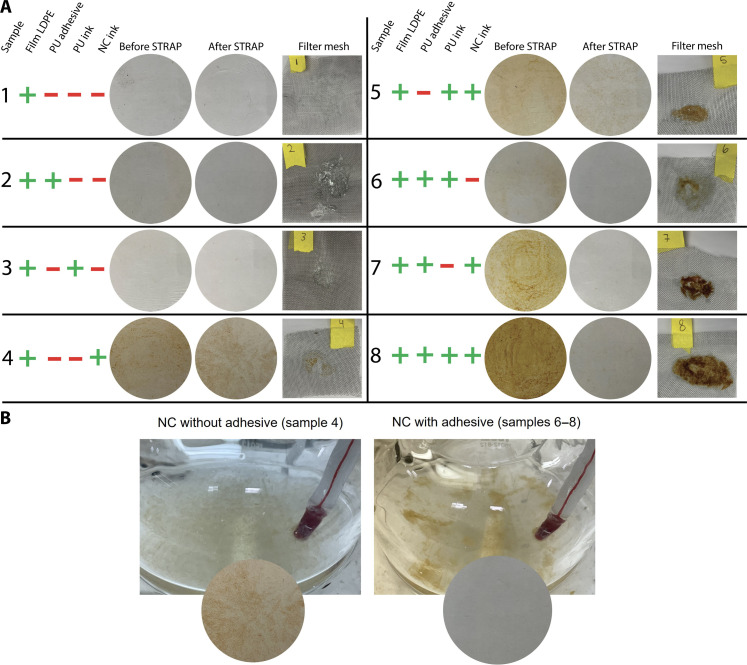
Compressed LDPE films containing adhesive and/or ink extenders before and after STRAP. The films included various formulations of unpigmented inks (extenders) and PU adhesives that are commonly used in printed multilayer films. (**A**) Sample formulations, appearance, and filter cakes. (**B**) Close-up pictures of solution with and without PU adhesive.

STRAP was applied to eight samples consisting of virgin LDPE plus various combinations of PU adhesive, PU ink, and/or NC ink extenders (ink binders, solvents, and additives, i.e., inks without pigments) to identify any possible degradation reactions that might cause yellowing during the STRAP process in the absence of pigments (Fig. 3). “Before STRAP” samples were hot pressed (method 1) at 190°C, while “After STRAP” samples were dissolved in dodecane at 95°C, filtered with a 600-μm filter, precipitated at 25°C, dried under vacuum at 110°C, and hot pressed (method 1) at 190°C. Compressed films of the samples before and after STRAP are shown in Fig. 3.

Sample 1 showed no yellowing before or after STRAP, indicating that degradation of the bulk polymer or any additives in the virgin resin is not the cause of yellowing. Slight yellowing was observed before STRAP for samples 2, 3, and 6, which is hard to see in Fig. 3. Samples 4, 5, 7, and 8 all show heavy yellowing before STRAP, indicating that NC ink causes this color change during the film hot compression. The PU adhesive and PU inks can also degrade but only form a slightly yellow color. Samples 2, 3, 6, 7, and 8 after STRAP showed no notable yellowing, while samples 4 and 5 after STRAP showed yellowing as in the pre-STRAP samples yet to a lesser degree. These results demonstrate that STRAP effectively reduced the yellow color of all samples caused by insoluble PU and NC. The reason for samples 4 and 5 yellowing is that some of the small NC particles passed through the relatively coarse filter in this experiment. The difference between samples 4 and 5 and samples 7 and 8 is that samples 4 and 5 did not contain the PU adhesive. The PU adhesive thus binds to insoluble agglomerates and other insoluble components to help filter off NC from the dissolved polymer (Fig. 3B). More importantly, the hue and heterogeneous dispersion caused by insoluble NC and PU do not match with the uniform green-yellow hue in our STRAP-recovered PE from printed films (Fig. 2B). Therefore, it can be reasonably assumed that none of the components in this experiment are the main source of yellowing in STRAP-recovered PE. This leads to the hypothesis that PE yellowing is likely caused by pigments in inks.

### Pigment selection and solubility measurements

An extended conclusion from the above STRAP experiments with adhesives and binders is that insoluble pigment particles can adhere to the ink binders and be removed more easily via filtration. Inorganic pigments exhibit extremely low solubility in STRAP organic solvents and may be targeted via filtration. Organic pigments are often partially soluble in STRAP solvents, making the color removal process more challenging. The following work focuses on methods to reduce the color caused by organic pigments in dissolution-based recycling.

**Fig. 4.**
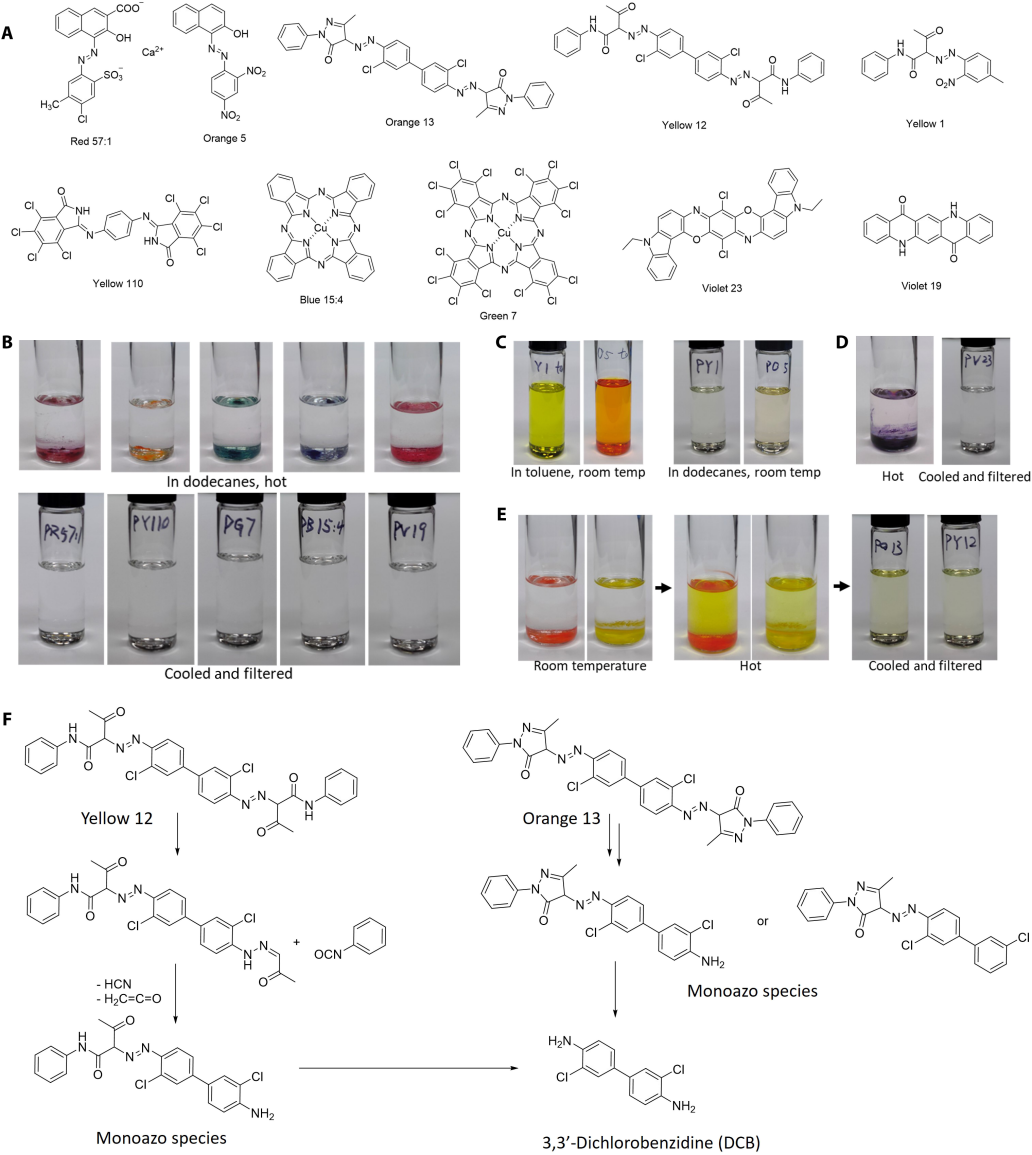
Pigments tested in groups by solubility in this study. (**A**) Chemical structures of pigments tested. (**B**) Red 57:1, Yellow 110, Green 7, Blue 15:4, Violet 19: insoluble at both 20° and 120°C. (**C**) Yellow 1 and Orange 5: soluble at 20°C. (**D**) Violet 23: insoluble at 20°C, soluble at 120°C. (**E**) Orange 13 and Yellow 12: insoluble at 20°C, soluble at 120°C, remain partial soluble upon cooling. (**F**) Possible decomposition route of diarylide pigments under elevated temperatures (*46*).

Organic pigments are frequently used in plastic packaging due to their higher color strength, brighter hues, and higher transparency, which can reduce costs compared to inorganic pigments (*6*). Yet, these pigments have lower solvent resistance against organic solvents than inorganic pigments. The industry continuously seeks pigments to achieve specific hues with single components and high color fidelity (*40*). A large array of chemical structures is available in synthetic pigments, and it is impractical to explore every variant’s property. In addition, ink manufacturers often keep their formulations confidential. Despite the vast number of individual organic pigments, common categories of organic pigments by chromophores include azo, phthalocyanine, quinacridone, and isoindolinone as shown in Fig. 4A (*6*, *18*, *41*, *42*). Azo pigments are broadly used in red, orange, yellow, and violet colors, and phthalocyanines are prevailing as blue and green pigments (*43*). Quinacridones and isoindolinones are more commonly applied in high-performance scenarios (*6*). Within each of these categories, the variants share similar structures leading to similar solubilities and, thus, are representative of properties of organic pigments in printing. Among the tested pigments, Yellow 12, Blue 15, and Red 57:1 are commonly used in the four-color (CMYK, i.e., cyan, magenta, yellow, and black) printing process (*8*).

Typically, during STRAP of the printed multilayer film samples, a yellow-green PE solution is collected after hot filtration. After precipitation and mechanical filtration, solvent and polymer are separated, although both still have an undesired color. Toluene produces more intensely colored PE while alkanes give a reduced yet recognizable yellowness (Fig. 2A) (*25*). As we will show here, the colorants that contaminate recovered PE are soluble but require heating and have higher solubility in aromatics than aliphatic solvents. Determining the exact source of this coloring is critical for understanding and resolving this issue in the STRAP process.

In Fig. 4, we categorized the tested pigments in groups by their dissolution behavior in dodecane (method 2). A large fraction of organic pigments (Red 57:1, Blue 15:4, Green 7, Yellow 110, and Violet 19) showed no visually notable solubility at both 20° and 120°C (Fig. 4B). The low solubility is caused by their stronger intermolecular interaction from large π-conjugated molecular structures, hydrogen bonding (Violet 19) (*44*), and/or ionic form (Red 57:1). As a result, no obvious contribution to PE yellowing is anticipated from this group of pigments. In the second group, Yellow 1 and Orange 5 are soluble at 20°C in both toluene and dodecanes (Fig. 4C), due to their low polarity and low molecular weight (*42*). Extracting the printed film samples with dodecanes under the same conditions resulted in no recognizable colorant dissolution as the solubility test, which indicates that our printed film samples do not contain these room temperature soluble monoazo pigments. This result agrees with the fact that monoazo pigments are incompatible with polyolefins due to bleeding and leaching issues (*6*). Dioxazine pigment (Violet 23) displayed conditional solubility (Fig. 4D), as it slightly dissolved in dodecanes at 120°C with a light color, while fully precipitated and removed upon cooling to 20°C and filtration. This matched the observations from STRAP experiments about solubility dependence on temperature. However, the dissolved color is relatively weak, and the blue-shade violet is the complementary color of yellow-green in the STRAP-recovered PE (*45*). Violet 23 is also not the main cause of PE coloring. Even if Violet 23 is present in the printed film and partially dissolves during STRAP, its color in the recovered PE will be masked by the dominating yellow-green colorant.

The dissolution behavior of diarylide orange and yellow pigments (Orange 13 and Yellow 12) is different from the other three groups. These two pigments showed no visually recognizable dissolution at 20°C, yet the dodecanes turned yellow-green after heating with the pigments at 120°C as shown in Fig. 4E. The difference between diarylide and dioxazine pigments is that Violet 23 remained insoluble in dodecanes upon cooling to 20°C, while the yellow color in hot solution of Orange 13 and Yellow 12 stayed at 20°C. Both the dissolution behavior and color match perfectly with our STRAP phenomena. Diarylide pigments also exhibit higher solubility in toluene than in alkanes (fig. S2), as we observed in Fig. 2A.

The yellow colors remained in the dodecane solutions of Yellow 12 and Orange 13 after cooling and filtration as shown in Fig. 4E while the pristine diarylide pigments are insoluble under the same temperature. This distinct solubility indicated that the dissolved coloring species have different chemical structures from the initial pigments. Literature suggests that diarylide yellow and orange pigments have lower thermal stability and decompose into monoazo species at high temperatures (above 200°C) as shown in Fig. 4F (*46*). These monoazo species have a higher solubility in organic solvents than the pristine diarylide species due to the reduced molecule sizes. A recent study on plastic recycling with inks confirmed through thermogravimetric analysis (TGA) that the decomposition of diarylide yellow occurs above 100°C (*8*), indicating the likelihood of forming these more soluble colorants under the STRAP conditions (*46*).

**Fig. 5.**
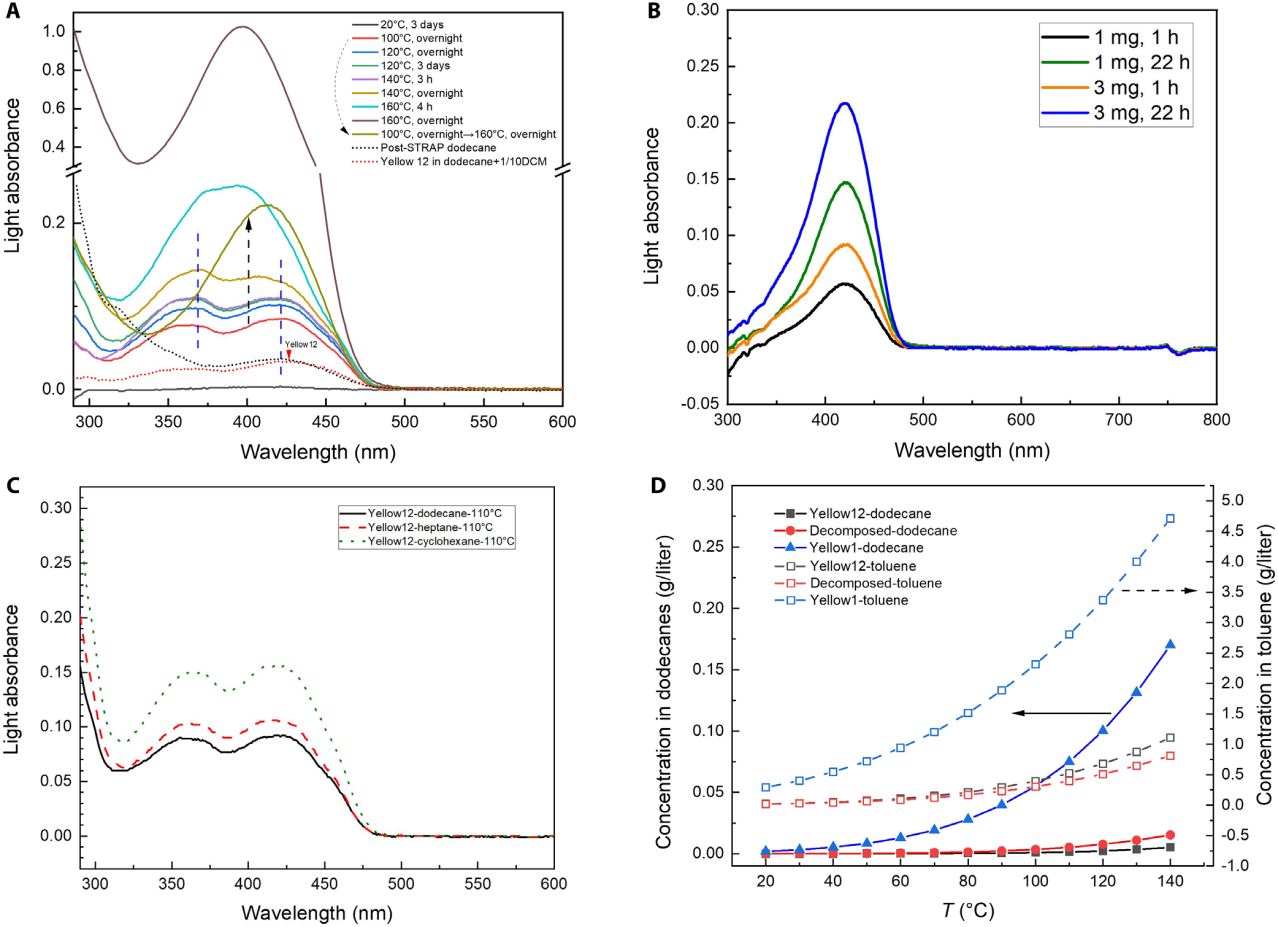
Further investigation of pigment Yellow 12 dissolution behavior. (**A**) UV-Vis of post-STRAP dodecanes and Yellow 12 solutions prepared at varied temperatures (1 mg/ml loading, filtered after cooling before UV-Vis measurement). (**B**) UV-Vis of Yellow 12 solution in dodecanes (10 ml solvent, 100°C, filtered after cooling). (**C**) Decomposed Yellow 12 solution in different alkanes (1 mg/ml loading, 110°C, 1 day, filtered after cooling). (**D**) Predicated solubilities of Yellow 12, decomposed Yellow 12, and Yellow 1 in dodecanes and toluene at different temperatures. Solubilities were predicted using a quantum chemistry–based equilibrium thermodynamics method, the conductor-like screening model for real solvents (COSMO-RS).

Ultraviolet-visible spectroscopy (UV-Vis) of the heated Yellow 12 solution in dodecane detected absorption peak shifting compared to the Yellow 12 solution prepared at 20°C (Fig. 5A), validating the molecular structural change of diarylide pigments upon heating. The maximum absorbance of heated (decomposed) Yellow 12 matched with the STRAP-recovered dodecanes used on the printed multilayer film (Fig. 5A), further supporting the hypothesis that diarylide derivatives contribute to PE yellowing.

Temperature plays an important role in colorant transformation. Raising the dissolution temperature from 20° to 100°, 120°, and 140°C increased the absorption peak at 420 nm with temperature and time as shown in Fig. 5A. The ratio of this peak over the other peak at 370 nm decreased with rising temperature. At 160°C, absorption increased drastically with a hypsochromic shift (Fig. 5A), suggesting accelerated formation and further structural evolution of soluble colorants. This change at 160°C aligns with reported TGA profiles of diarylide pigment, in which its first decomposition peak is at 162°C (*8*). After removing undissolved pigments, heating the yellow dodecane solution at 160°C still resulted in a noticeable increase in absorbance with a hypsochromic shift, demonstrating that dissolved color species also undergo changes at high temperatures (Fig. 5A, black arrow).

The color of dodecane solutions prepared by heating diarylide yellow pigments also depends on the pigment loading and heating time. Increasing the Yellow 12 loading from 0.1 to 0.3 mg/ml or extending the heating time increases the solution light absorbance as shown in Fig. 5B. If the concentration of dissolved species is governed solely by dissolution equilibrium, the color should depend only on temperature (*47*). However, experimental evidence reveals that yellowing during dissolution-based PE recycling involves both pigment dissolution and pigment decomposition, the latter of which depends on more factors.

Quantitative analysis of the solubility of the decomposed pigment was performed using UV-Vis. While the exact molar masses or chemical structures of dissolved colorants from diarylide pigments remain unknown, their solubilities can be estimated by comparing their absorbance in solution to known pigments (e.g., Yellow 12 and Yellow 1). The molar absorptivity of Yellow 1 is on par with Yellow 12 (fig. S2) due to the structural similarity. Therefore, we assume that decomposed and intact Yellow 12 also share similar absorptivities because they have similar chemical structures.

The solubility of Yellow 12 was measured using UV-Vis (fig. S2): 25 mg/liter (20°C, toluene), negligible (20°C, dodecanes), and 0.3 to 1.2 mg/liter (120°C then 20°C, dodecanes, equivalent to the dissolved species in solution, varying with conditions). Yellow 1 is more soluble than Yellow 12 with a solubility in dodecane of 2.1 mg/liter at 20°C. Given the structural similarity between Yellow 1 and diarylide decomposition products, it is estimated that these presumed soluble colorants have similar solubilities as well. It was observed that decomposed diarylides sometimes reached saturation during dissolution tests at elevated temperatures with high pigment loadings, agreeing with our hypothesis that the solubility of decomposed pigments is still limited. The absolute content of dissolved yellow compounds is low, although the color is obvious due to high molar absorptivities.

The solubility of colorants in heptane and cyclohexane was also measured (Fig. 5C). These solvents have lower boiling points and could be energy-efficient alternatives to dodecane in STRAP. Cyclohexane dissolves 70% more Yellow 12 than dodecane. Heptane only dissolves 15% more colorants from Yellow 12 than dodecanes. The conductor-like screening model for real solvents (COSMO-RS) was used to predict the solubility in different solvents (Fig. 5D). The solubilities of arylide yellow, diarylide yellow, and proposed decomposition species were quantitatively predicted in different solvents based on density functional theory (DFT) calculations of charge density distribution and physical properties (melting point and enthalpy of fusion). The chemical structure of decomposed Yellow 12 used in the calculation was the monoazo species from Fig. 4F. Although the predicted solubility of decomposed Yellow 12 did not match perfectly with experimental data (likely due to the complex nature of decomposition reactions), the computational prediction indicated heavy dependence of colorant solubility on temperature (Fig. 5D), suggesting that the diarylide dissolution without decomposition is also not negligible. In Fig. 4E, the hot solution’s color was visually deeper than the cooled solution, suggesting that color change in the liquid phase resulted from both diarylide decomposition and hot dissolution, which agrees with the COSMO-RS results.

In general, the coloring of STRAP-recovered polymers is mainly due to dissolution and decomposition of diarylide pigments. The pigments we tested represent the majority of pigment categories used in packaging printing processes (*8*). Alkanes are commonly chosen as selective solvents for polyolefin recovery in dissolution-based approaches (*5*, *48*). The solubility is highly dependent on conditions such as temperature, pigment content, and dissolution time. The dissolved yellow compounds at elevated temperatures pass through hot filtration along with solvents and dissolved polymers. Some of the derived species remain soluble after cooling to 20°C. To our knowledge, this combined process of pigment dissolution and decomposition has not been reported in dissolution-based recycling. The results here provide a general explanation that the color (yellowness) of resins recycled by dissolution-based methods (*19*, *33*), even when the mixed plastic sources consist of all colors of pigments (*19*, *33*), is due to the diarylide pigments and decomposition of the diarylide pigments into monoazo compounds.

### Factors contributing to plastic coloring

We can now analyze colorant accumulation during the STRAP process thanks to our understanding of pigment behavior. This section identifies the major factors contributing to the final coloring of the polymer product and proposes solutions to mitigate this issue. We refer the reader to Fig. 1 to highlight the key process steps during this discussion.

First, despite the obvious color from dissolved colorants, the decomposed ink solubilities are relatively low (<4 mg/liter, even under elevated temperature). In the printed multilayer packaging films, the ink content is typically below 5 wt % of the total packaging mass, usually ranging from 2 to 4 wt % (*8*, *39*). Printing inks generally contain 5 to 50 wt % pigments (*49*). Given that our printed film samples are mostly yellow to orange, a conservative estimate is that yellow pigments constitute approximately 50 wt % of the total pigments. Thus, the printed packaging samples in our experiments are estimated to contain 0.05 to 0.6 wt % yellow pigments before STRAP.

In a typical STRAP process, the solvent-to-plastics ratio is set to be 10:1 by mass (*5*, *25*, *28*–*30*). Consequently, the total mixture in the dissolution tank contains 50 to 600 parts per million (ppm) of yellow pigments, while the dissolved fraction is less than 5 ppm pigments at elevated temperature according to the solubility. Therefore, the majority (>90 wt %) of yellow pigments remain insoluble during the STRAP dissolution step and are removed in stream 3 (Fig. 1) during hot filtration. Yet, this small amount of pigment will still cause a yellow color in the recovered plastic.

After hot filtration, the filtrate (stream 4 in Fig. 1) contains dissolved PE and colorants as a homogeneous solution. When this solution cools during the precipitation step, PE is almost completely precipitated and separates from the solvent phase, while the colorants distribute in different phases. Intact Yellow 12 molecules coprecipitate with PE because of their low solubility at 20°C. In contrast, the pigment decomposition products remain (partially) soluble in the solvent at 20°C.

The colorants in the precipitated mixture are further partitioned in the following mechanical separation step. Precipitated colorants remain with PE in stream 8, while the dissolved fraction is mostly separated into the filtrate solvent (stream 7), resulting in a yellow recovered solvent. However, the polymer cake leaving the mechanical separation step (stream 8 in Fig. 1) can contain up to approximately three times more solvent than PE by mass, which also carries dissolved pigment decomposition products. This retained solvent, along with the precipitated colorants, contributes to the yellowness of recycled PE via STRAP. Improving the mechanical separation efficiency could help remove colorants from the precipitated PE by reducing solvent retention.

During the drying step, only the solvent is removed, typically by vacuum. The remaining (di)arylide colorants are not volatile (*8*) and are left behind with the PE. Last, dried PE is pelletized via extrusion at 190°C, which potentially leads to further decomposition of the colorants and further discoloration (Fig. 2B). The main factors determining the color in recycled PE are as follows: (i) the concentration of the dissolved pigment in the solvent and (ii) mechanical separation efficiency of solvent from precipitated PE.

Decreasing the temperature and reducing heating time while ensuring efficient PE dissolution are two strategies to minimize the dissolved decomposed colorant concentration. Another option is to remove more solvent during the mechanical separation step shown in Fig. 1. This can be achieved by using an advanced filtration approach like centrifugation or compression filtration to remove solvent from the polymer-solvent cake, thus reducing the colorants in the PE after drying (streams 8 to 11).

In addition, adsorption of the pigments in the hot PE solution (streams 4 to 6) before precipitation should also decrease the colorant concentration in the solvent and even prevent colorant precipitation during cooling. The two approaches can be deployed concurrently to minimize the residual colorants in the STRAP-recovered PE. In the following sections, we will discuss the pigment removal efforts in detail.

### Compression filtration: Reduce solvent retention

In previous STRAP experiments (*5*, *25*, *28*–*30*), the filtration step after PE precipitation was completed via vacuum filtration as shown in fig. S3. The PE product was a cake with approximately 75 wt % of it being dodecane. We modified the mechanical filtration system by adding a pneumatic piston to apply compressive force as shown in fig. S3 to further deliquor the PE filter cake. The force of the pneumatic piston can be controlled with a pressurized gas.

**Fig. 6.**
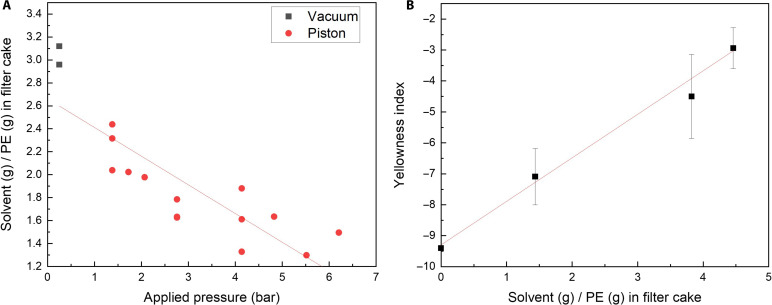
The impact of mechanical deliquoring on solvent and color removal. (**A**) Ratio of retained solvent mass to PE mass in filter cake versus applied pressure. (**B**) PE yellowness versus solvent retention. LDPE precipitated from dodecane solution with 0.86 mg/liter decomposed Yellow 12, with different amount of dodecane solution in the filter cake. The error bars indicate the SD caused by measurement and sample thickness inhomogeneity.

PE cakes were produced with varying pressures to document the effect of mechanical compression on solvent extraction using the compression filtration system shown in fig. S3. The results are plotted in Fig. 6A, which compares the deliquoring results via vacuum filtration and piston compression filtration. The variation seen in Fig. 6A can be attributed to the nonhomogeneous nature of the precipitated slurry. A single STRAP experiment is broken into multiple deliquoring batches, each with slightly varied composition of PE and solvent. Compression effectively reduced the solvent-to-PE ratio in the filter cake from 3.1 to 1.3 without altering the PE recovery ratio from printed films. We observe a clear inverse correlation between pressure and solvent-to-PE ratio in filter cake.

A series of small-scale experiments were performed to evaluate the impact of solvent removal on color (method 3). Instead of adding Yellow 12 pigments, a premade dodecane solution of decomposed Yellow 12 was mixed with commercial LDPE at 100°C and stirred. Upon cooling, the mixture was transformed into a slurry. The solvent was removed by compression and vacuum filtration to keep track of the solvent-to-PE ratio of the filter cake. The cake was then dried in a vacuum oven and the LDPE was converted into a compressed film. The results, shown in Fig. 6B, reveal a clear positive linear trend between solvent retention and PE yellowness. Yellowness index (YI) is a common industry metric used to describe the yellowness of a resin or film; lower values correlate to less yellowness, and a virgin resin may have a YI of approximately −10 under the measuring conditions. The YI of our recovered LDPE decreased from −2.94 to −7.09 as the amount of solvent retained in the filter cake decreased from 4.5 g of solvent per gram of PE to 1.4 g of solvent per gram of PE. Thus, final PE yellowness was directly proportional to the solvent retention in the polymer filter cake. Considering the improved efficiency of solvent removal, mechanical deliquoring is preferable to vacuum filtration in the STRAP color removal process.

The total amount of colorant in the filter cake comes solely from trapped solvent, assuming colorants have negligible solubility in the precipitated PE polymer phase. This amount is estimated by multiplying the colorant concentration in the solvent by the volume of solvent in the cake. Subsequent solvent evaporation of the filter cake removes only the solvent, not colorants. Therefore, the final colorant content (*Color*_*PE*_) in the dried LDPE can be calculated using Eq. 1 in which *C*_solv_ is the soluble colorant concentration in the solvent (*m*/*V*) determined by UV-Vis, where *V*_solv_ is the solvent volume in the filter cake, given by (*m*_cake_ – *m*_PE_)/ρ_solvent_; *m*_cake_ is the mass of filter cake, m_PE_ is the mass of dried PE

$${\text{Color}}_{\text{PE}}=C_{\text{solv}}V_{\text{solv}}/m_{\text{PE}}$$ (1)

The observation that reducing solvent retention via compression leads to a proportional decrease in colorant levels in PE aligns well with experimental results. This finding offers a useful method for estimating recycled polymer color based on the weight of the filter cake and the absorbance of the recovered solvent. In addition, by accounting for the coprecipitation of undecomposed diarylide pigments, we obtain the following modified expression for colorant levels shown in Eq. 2 where $C\prime_{\text{sat}}$ is the undecomposed pigment (e.g., Yellow 12) solubility in hot solvent (*m*/*V*) and *V*_total_ is the total volume of solvent used during the dissolution step. We assume that the undecomposed pigment has minimal solubility in cooled solvent, so all the dissolved intact diarylide pigments $\left(C\prime_{\text{sat}}*V_{\text{total}}\right)$ coprecipitate upon cooling

$${\text{Color}}_{\text{PE}}=\left(C_{\text{solv}}V_{\text{solv}}+C\prime_{\text{sat}}V_{\text{total}}\right)/m_{\text{PE}}$$ (2)

Moreover, given that the colorant level in dried PE is determined by both the solvent content in the filter cake and the pigment concentration in the solvent, reducing the colorant concentration in the solvent should also decrease the YI in the recovered PE. This is verified by experimental results (fig. S4), in which the YI followed the same linear trend from Fig. 6B with changes to both solvent retention and concentration of pigments in the solvent. By using compression filtration and reducing soluble colorant concentration, the YI of recovered PE can approach that of virgin PE.

### Adsorption: Reduce dissolved colorant concentration

We can expect to reduce the color in the final product resins by reducing the concentration of dissolved colorants in the hot polymer solution before precipitation. Adsorption of the dissolved contaminants is a potential solution that has been applied to remove colorants in industrial plastic recycling processes (*50*). For example, an adsorption bed is deployed in the PureCycle process of PP purification for color removal (*50*). With the knowledge of the color source in the solvent and polymers, we will now begin to quantify the ability to remove colorants in STRAP via adsorption.

**Fig. 7.**
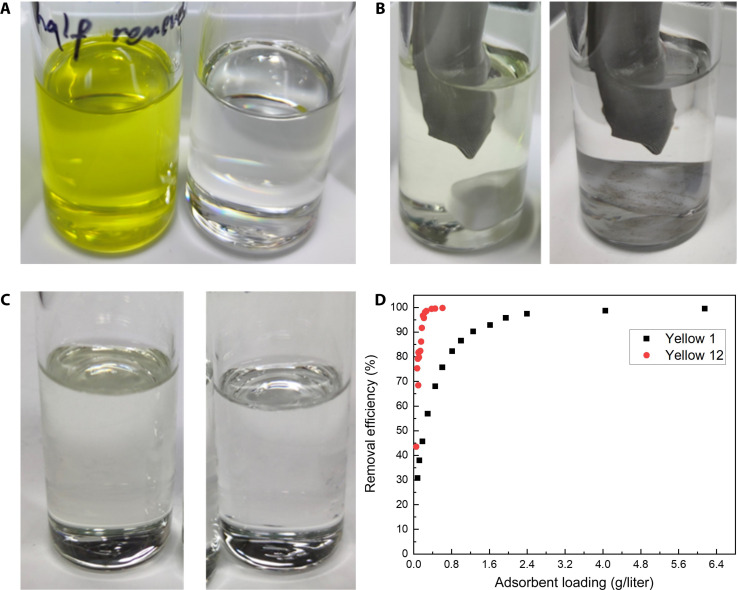
Comparison of pigment solutions before (left) and after (right) adsorption with activated carbon at 20°C. (**A**) Yellow 12 toluene solution. (**B**) Decomposed Yellow 12 dodecane solution, heated then cooled and filtered before adsorption. The AC is contained in a mesh bag, seen in the photos. (**C**) Post-STRAP dodecanes with dissolved yellow colorants. (**D**) Color removal efficiency versus AC loading in 25 ml of Yellow 12 (15 mg/liter) or Yellow 1 (16 mg/liter) toluene solution. Color removal efficiency versus AC loading in 25 ml of Yellow 12 (15 mg/liter) or Yellow 1 (16 mg/liter) toluene solution.

Qualitative experiments at 20°C, shown in Fig. 7, demonstrate that activated carbon (AC) effectively adsorbs dissolved colorants, turning solutions of Yellow 12 and its decomposition products into colorless solvents. AC also successfully removed yellow color from post-STRAP dodecanes.

The adsorption efficiency of pigments by AC was measured (Fig. 7D) with Yellow 12 and Yellow 1 as the model molecules in toluene at 20°C to better understand adsorption behavior. The reason for not applying higher temperatures or using dodecanes in the quantitative study is due to the pigment decomposition and the fact that pigment solubility in dodecane at room temperature is too low for quantitative analysis of adsorption. The efficiency increases with the adsorbent dose as expected, due to the increase in total adsorbent surface area and available adsorption sites. The increase in removal efficiency is the steepest at the beginning of the curves and flattens out when the maximum value of 100% is approached (Fig. 7D). This can be explained by the decrease in concentration gradient of the adsorbate molecules between the solid and liquid phase as adsorption occurs, causing a reduction in driving force. A removal efficiency of 90% was achieved at an adsorbent dose of approximately 0.17 and 1.26 g/liter for Yellow 12 and Yellow 1, respectively, while an efficiency of 99% requires approximately 0.31 and 4.4 g/liter.

A makeshift adsorption bed (fig. S5) was used to verify pigment removal from solvents in a continuous system. With an average weight hour space velocity (WHSV) of 100 to 150 hour^−1^, AC adsorbent can completely decolorize more than 860 times of test pigment solution (Yellow 12 in toluene, 7 mg/liter) by mass (150 mg AC versus 150 ml solution), before loss of adsorption efficiency. The adsorption capacity of the bed was lower than batch experiments but still of the same order of magnitude.

Solubility measurement results suggest that (di)arylide pigments have lower solubility in alkanes; therefore, AC is anticipated to exhibit improved adsorption capacities in dodecanes than toluene. An even higher removal efficiency can be expected to counterbalance the reduced driving force (concentration gradient) in post-STRAP dodecanes. Moreover, post-STRAP dodecanes contain less colorants, allowing the adsorption bed to serve longer before replacement.

**Fig. 8.**
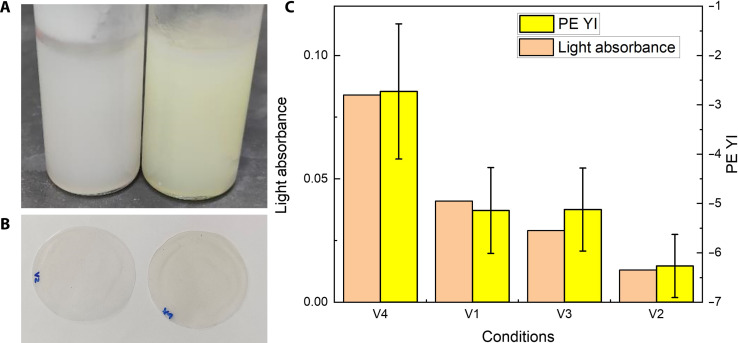
Hot adsorption experiment with PE solution. PE (1.2 g) was dissolved in 36 ml of premade decomposed Yellow 12 and Orange 13 dodecane solution. AC pellets were added in mesh cages (for effective removal afterward) and stirred before cooling and filtration. PE weight fraction in the cake is controlled at 30% via vacuum filtration and compression before drying. (**A**) Cooled PE solution with (left) and without (right) adsorbent treatment. (**B**) Hot-pressed LDPE film samples, V4 (left, no adsorbent) is yellower; V2 (right, 200 mg AC, 18 hours of treatment) looks the least yellow. (**C**) YI of PE versus filtrate solvent UV-Vis absorbance. V1: 300 mg AC, 4 hours of treatment; V2: 600 mg AC, 18 hours of treatment; V3: 600 mg AC, 4 hours of treatment; V4: no adsorbent, control group.

Preliminary hot adsorption experiments were conducted according to method 5 with premade mixed solutions of decomposed Yellow 12 and Orange 13 and AC pellets. The results are summarized in Fig. 8. Visually, after hot adsorption and precipitation, the PE-dodecane-mixture looked white, while the control sample without adsorption was obviously yellow (Fig. 8A). The hot-pressed films from the recovered PE also displayed more yellowness in the control sample than hot-adsorbed PE (Fig. 8B).

Furthermore, the colors of PE films and filtrate dodecane solvents were quantitatively measured by a spectrophotometer. In agreement with the discussion of the relationship between dissolved colorants and color of recycled PE, the YI and filtrate absorbance followed the same trend alongside the adsorption progress and exhibited reduced yellowness (Fig. 8C).

When applying hot adsorption to real waste plastic such as a multilayer printed film, it is important to remove any large insoluble polymers or contaminants before the hot adsorption step. If the printed flakes are not removed first, they will continue to leach more colorants into the solution as the AC adsorbs colorants, maintaining the equilibrium concentration of colorants in solution.

Reducing the solvent in the filter cake and adding hot adsorption are two independent but complementary methods for color reduction in the recovered resins. Our results indicate the reduction of solvent retention and hot adsorption operate independently, with their effects being multiplicative in enhancing color removal. Thus, a STRAP experiment with modified setup and procedure was conducted in pursuit of obtaining colorless recycled polymer resins.

### Large batch validation

**Fig. 9.**
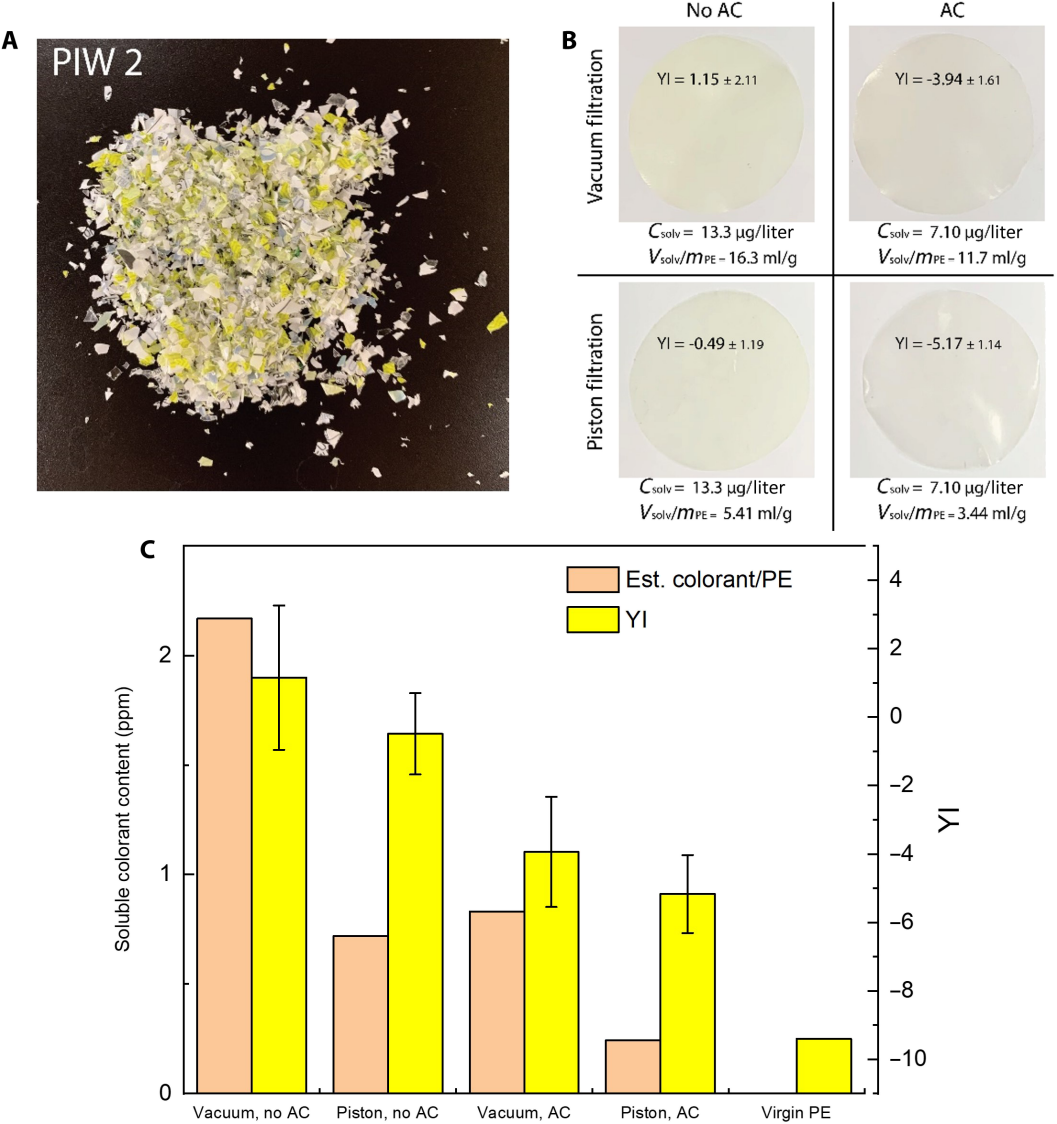
Large-batch color removal from reverse-printed laminated film sample (PIW 2). (**A**) PIW 2 film is primarily printed with green, white, yellow, and black. (**B**) PE was recovered from a single PIW feedstock, and a comparison of color removal methods is displayed. In addition to yellowness (YI) of the films, the concentration of pigments in the recovered solvent and the solvent retention (*V*_solv_/*m*_PE_) in the deliquored polymer cake are displayed. (**C**) Estimated soluble colorant concentration via Eq. 1 and YI of films are plotted for each of the four batches of PE.

A larger-scale experiment was performed with a different batch of postindustrial reverse-printed laminated film sample (PIW 2) to show that our findings can be generalized across printed films. PIW 2 contained PE, PET, EVOH, PU, and inks, like PIW 1 (used previously at small scale), but the specific formulation and thickness of each layer is unknown. PIW 2 is also a different color than PIW 1 (Fig. 9A). These experiments treated ~150 g of PIW in a single batch, representing a scaleup factor of approximately 300× when compared to previous color removal tests. In two identical batches, PE was extracted from the PIW with dodecane. After dissolution, one batch was immediately precipitated while the other was further treated with AC before precipitation. After precipitation of both batches, each batch was split in half and the solvent was recovered by either vacuum filtration or piston compression, before oven drying. Figure 9B displays the corresponding hot-pressed films.

Overall, the same trends previously observed with color removal in PIW 1 hold true for PIW 2. Both mechanical deliquoring and hot solution adsorption were effective in reducing the yellowness in comparison to the conventional STRAP setup with vacuum filtration. The final PE recovered from PIW 2 by using both color reduction methods had a YI of −5.2 without further optimization, which is slightly yellower than the reprecipitated PE from colorant solutions (ranged from −6 to −7) in model experiments (Figs. 6 and 8), due to the more complicated film composition in real PIW than the model experiments above.

According to Fig. 9B, yellowness was reduced with a decrease in solvent retention in the filter cake (*V*_solv_/m_PE_) as expected. Solvent retention was higher in PIW 2 than in PIW 1, which is a likely contributor to higher yellowness in the PE recovered from PIW 2. Factors such as molecular weight and branching (crystallinity) likely affect the solvent retention behavior of polyolefins. This topic is beyond the scope of this paper, but worth investigation in future research.

Adsorption reduced the yellowness of the recovered PE. Comparing the UV-Vis absorption of the recovered solvent from the AC-treated batch and the untreated batch, the dissolved colorant concentration (*C*_solv_) was reduced by approximately 47% after adsorption (Fig. 9C), agreeing with the smaller-scale experiment in Fig. 8C. The YI decreased by 4.7 and 5.6 with and without piston compression, respectively. In comparison to Fig. 8C and fig. S5, the PE recovered from PIW 2 is slightly yellower than expected. In Fig. 9C, the decrease of yellowness in the samples treated by piston deliquoring was not as much as anticipated based on the soluble colorant contents. We believe that soluble, undecomposed pigments that coprecipitate with the polymer may be the cause of these discrepancies. As discussed in Eq. 2, undecomposed diarylide pigments almost completely coprecipitate with PE, and this fraction of yellowness is not affected by deliquoring. Nonetheless, both adsorption and solvent removal techniques contributed to substantial decrease of color in rPE recovered from a colored multilayer waste.

## DISCUSSION

In this paper, we outline how the STRAP process can remove color from reverse-printed laminated multilayer PE films. We learned that decomposed diarylide pigments are the main source of yellowness in STRAP-recovered resins, and this is likely true for other solvent-based recycling methods as well. The solubility of these pigments is a function of the solvent and temperature. Diarylide pigments have a lower solubility in alkane solvents like dodecane than in aromatic solvents like toluene. UV-Vis can be used to quantify the amount of dissolved pigments in a solvent and to predict the color of the resulting recovered polymer in a STRAP process. Moreover, the retained solvent in the resin filter cake with decomposed soluble colorants directly contributes to the resin color.

Our finding is not limited to a specific feedstock or polymer type. A general methodology was developed for color removal from recycled polymer resins. The first step in reducing the color of recycled plastics is to identify solvents in which the pigments have low solubility. However, it is unlikely that the colorants will be completely insoluble; thus, two additional approaches are needed to remove the color from STRAP films. First, mechanical filtration must be optimized to reduce solvent retention in the precipitated polymer before thermal drying. Second, the colorants need to be removed from the solvent. This removal can be applied to the hot dissolved solution before precipitation as well as the cold recycled solvent and is preferably done by adsorption.

The aforementioned color reduction methods were successfully applied to the STRAP process to recover PE with color similar to virgin PE. This proves that colorants are carried by the solvent and reducing both colorant concentration in the solvent and solvent retention in the precipitated polymer are key to obtaining a colorless recycled plastic product. This also indicates that solvent-based recycling methods such as STRAP have the potential to produce high-quality recycled resins, thereby improving the circularity of printed multilayer plastic packaging.

Future work should focus on verifying the versatility of our developed approaches. Our color removal method is based on the understanding of pigment behavior during the STRAP process, which suggests its potential in broader plastic recycling scenarios. Recovering other polymer components from reverse-printed laminated multilayer films and other waste plastic feedstocks will grant us more detailed knowledge of behaviors about colorants and similar contaminants during varied STRAP conditions. This knowledge will help us validate and improve the adaptivity of our approach. This is critical due to the high complexity of plastic wastes. Together with the simple unit operation, the high adaptivity of our color removal approach make the STRAP process a promising strategy toward higher-quality recycled polymer resins and reduction of plastic wastes.

## MATERIALS AND METHODS

### Materials

The printed flexible multilayer films (PIW 1 and PIW 2) were collected from an Amcor postindustrial waste stream, consisting of a PE layer, an EVOH layer, a PU-based adhesive layer, a PU-based ink layer, and a PET layer. The materials were shredded through a 1/8″ cross-cut shredder (Make: Allegheny 16-75CX) at Michigan Technological University. The pure virgin LDPE resins were received from Amcor. LDPE (EG412, Westlake), dodecanes (mixture of isomers, technical, Thermo Fisher Scientific), AC powder (SX1G, Norit), AC pellets (R3Extra, Norit), Pigment Red 57:1 (lithol rubine BCA, >85.0%, TCI), Pigment Orange 5 (95%, AstaTech), Pigment Orange 13 (pyrazolone orange, TCI), Pigment Yellow 12 (Santa Cruz Biotechnologies), Pigment Yellow 1 (Hansa Yellow, AstaTech), Pigment Yellow 110 (Santa Cruz Biotechnologies), Pigment Blue 15 [copper(II) phthalocyanine, Thermo Fisher Scientific], Pigment Green 7 (TCI), Pigment Violet 19 (quinacridone, >93.0%, TCI), and Pigment Violet 23 (AstaTech) were received from suppliers without further purification.

### Method 1—Hot pressing LDPE films

Small films for color testing were produced by placing 0.75 g of polymer between two 6″ × 6″ polytetrafluoroethylene (PTFE)–coated fiberglass sheets followed by two 6″ × 6″ aluminum sheets on the outside. A Carver Press 3970 was used to heat the sample at 190°C without pressure for 1 min, followed by 3 metric tons of pressure for 1 min.

### Method 2—Pigment dissolution test

In a 30-ml scintillation vial, 10 mg of organic pigment, 10 ml of dodecane, and a magnetic stir bar were added. The scintillation vial was then sealed and placed in an aluminum heating block. The system was heated to the desired dissolution temperature with an electric heat plate equipped with a magnetic stir drive, and the stirring rate was adjusted to have constant mixing. After the preset heating time, the heating was stopped, and the system was allowed to cool to 20°C. The mixture was filtered through a 0.22-μm PTFE syringe filter. The filtrate was collected for UV-Vis measurement.

A calibration curve of Yellow 12 was obtained by measuring UV-Vis absorbance of standard solutions of Yellow 12 with varied concentrations. The absorbance of saturated Yellow 12 solution was measured and fitted to the extrapolated calibration curve to determine pigment solubility.

### Method 3—Investigating the relationship between solution retention and PE yellowness

Decomposed Yellow 12 solution was prepared by stirring Yellow 12 pigment in dodecanes (1 g/liter) at 120°C for varied reaction times, then cooled to 20°C and filtered to remove undissolved pigments. The absorbance and concentration of resultant solutions were determined from UV-Vis calibration.

LDPE (1.1 g) was dissolved in 20 ml of premade solution of decomposed Yellow 12 at 100°C for 2 hours, then cooled to 20°C. The hot solution turned into a yellowish gel upon cooling. The gelled solution was vacuum-filtered with compression. The filter cake was then transferred into a scintillation vial with the weight of filter cake recorded, followed by vacuum drying in a vacuum oven at 100°C for 3 hours. The weight of dried LDPE was recorded again before hot pressing the LDPE into films and measuring the YI.

### Method 4—STRAP vacuum filtration and compression filtration

During vacuum filtration, a Buchner flask and funnel were connected to a vacuum pump (model). Whatman 1114-150 Quantitative Filter Paper (25 μm) was used on top of the Buchner funnel. The precipitated polymer slurry was filtered until solvent flow stopped (fig. S3).

Piston compression was performed in a custom system (fig. S3) comprising the following key items from BVV: a 600X6FJ spool, an HRSJ600 reducer stand, an FP600-V3 filter plate, an SD1M-6B 1-μm filter, and a custom machined aluminum piston. The vessel was filled with ~1.2 liters of precipitated polymer slurry above the 1-μm filter, the piston was placed above the slurry, and the vessel was closed and pressurized. Filtration occurred until the solvent flow stopped.

### Method 5—Hot adsorption experiments

In a typical hot adsorption experiment, 1.0 g of LDPE was dissolved in 12 ml of decomposed Yellow 12 solution in dodecanes at 100°C with magnetic stir bar agitation. After complete dissolution, 100 mg of AC pellets was loaded in a metal mesh cage and immerged into the PE hot solution. The mixture was agitated for a certain period of time, then the mesh cage was lifted from the PE solution to remove adsorbent. The PE solution was cooled to 20°C and transformed into slurry due to PE precipitation. The slurry was vacuum-filtered with compression to collect the PE filter cake. The filter cake was then transferred into a scintillation vial with the weight of filter cake recorded, followed by vacuum drying in a vacuum oven at 100°C for 3 hours. The weight of dried LDPE was recorded again before hot pressing the LDPE into films and measuring the YI.

### Method 6—Color quantification

The UV-Vis absorbance and spectra of organic pigments solution were measured on a Thermo Fisher Scientific Evolution 300 UV-Visible Spectrophotometer with 10-mm quartz cuvettes. Calibration of UV-Vis spectrophotometer was completed with Yellow 12 solution in toluene as the standard (dissolving 7.0 mg Yellow 12 in 1.00 liter of toluene). The standard solution was further diluted with toluene or dodecanes to collect calibration data points. The final color of the recovered polymers was quantified with a spectrophotometer, and measurements were converted to CIELAB color space values, L* (lightness), a* (red-green color component), and b* (blue-yellow color component), and YI (*51*). For detailed characterization information, see the Supplementary Materials.

### Computational modeling

Following our previous work, we used the COSMO-RS to identify solvents for STRAP (*5*, *29*, *30*). COSMO-RS uses statistical thermodynamic methods to compute the equilibrium properties of multicomponent systems based on the screening charge density that arises at each compound’s molecular surface due to the polarization of the medium (*52*, *53*). Screening charge density profiles were obtained from DFT calculations performed using Gaussian 16 at the BVP86/TZVP/DGA1 level of theory (*54*–*56*). Screening charge densities were then used to predict polymer solubilities via a solid-liquid equilibrium calculation using the COSMOtherm 19 software with the BP_TZVP_19 parameterization (*30*, *57*, *58*). This solubility calculation requires the pigment melting temperature as input. It also involves the estimation and calibration of Gibbs free energy of fusion of the pigments based on quantitative structure-property relationship models and an experimentally measured solubility (fig. S3) in a reference solvent. Detailed computational information is available in the Supplementary Materials.

## Supplementary Materials

### This PDF file includes:

Supplementary Text Sections S1 to S5 Figs. S1 to S5 Tables S1 and S2
