# Supplementary material for: Pigment removal from reverse-printed laminated flexible films by solvent-targeted recovery and precipitation
Source: Sci Adv. 2025 Mar 14;11(11):eadt5841. doi: 10.1126/sciadv.adt5841 (PMC11908474; doi:10.1126/sciadv.adt5841)
Supplement: Supplementary file 1 — Supplementary Text Sections S1 to S5 Figs. S1 to S5 Tables S1 and S2 [file sciadv.adt5841_sm.pdf]

Science Advances
AAAS

Supplementary Materials for

Pigment removal from reverse-printed laminated flexible films by solvent-targeted recovery and precipitation

Tianwei Yan *et al.*

Corresponding author: George W. Huber, gwhuber@wisc.edu

Sci. Adv. **11**, eadt5841 (2025)
DOI: 10.1126/sciadv.adt5841

This PDF file includes:

Supplementary Text
Sections S1 to S5
Figs. S1 to S5
Tables S1 and S2

S1 Information about common pigments used in packaging printing process

Table S1 and fig. S1 display the common pigments and the corresponding chromophores (in organic pigments only). This information is utilized for selecting pigments in the dissolution behavior tests.

S2 Solubility of arylide and diarylide pigments via experimental method and calculation

UV-Vis calibration and measurement of pigments

Standard solutions of Pigment Yellow 12 and Pigment Yellow 1 were prepared and diluted to different concentrations to plot the UV-Vis calibration curve shown in fig. S2.

Saturated pigment solution was prepared and filtered to remove excess pigments. The solution sample was collected and diluted to ensure the concentration fell in the calibrated range before UV-Vis measurement. The solubility was obtained via fitting the measured absorbance with the corresponding calibration curve.

COSMO-RS calculation

The conductor-like screening model for real solvents (COSMO-RS) predicts thermodynamic properties of multicomponent systems based on quantum mechanical calculations and statistical thermodynamics methods.(*52, 53*) COSMO-RS represents each molecule based on the screening charge density that arises at its molecular surface due to the polarization of the medium. In this work, the screening charge density profiles of the solvents are obtained from COSMObase-1901-BP-TZVP, and the profiles of pigment molecules are acquired via density functional theory (DFT) calculations with the Gaussian 16 software package.(*55*) In the DFT calculations, we first performed geometry optimization in an implicit solvent using the conductor-like polarizable continuum model (CPCM) and BVP86/TZVP/DGA1 level. Then a single point calculation at the same DFT level was used to obtain screening charges in the infinite dielectric constant limit. These screening charge profiles serve as the input to the COSMOtherm 19 software with the BP_TZVP_19 parameterization to estimate the Gibbs free energy of fusion of pigments and predict pigment solubilities in various solvents.(*57, 58*) The COSMOtherm calculations require the melting temperature of the pigments as input parameters. The melting temperature of Pigment Yellow 12 is 320 °C based on the OECD Existing Chemicals Database. The data for the decomposed yellow 12 are unavailable, thus an estimated melting temperature of 256 °C was used based on the similar molecule Pigment Yellow 1. In COSMOtherm calculations, we first estimate the Gibbs free energy of fusion, ΔG_{fus}, of the pigment at room temperature using an embedded quantitative structure-property relationship (QSPR) method:

$$-\Delta G_{fus} = c_1\mu_w + c_2N^{ring} + c_3V + c_4$$

where c_1 to c_4 are the QSPR parameters, μ_w is the chemical potential of the molecule in water, N^{ring} is the number of ring atoms, and V is the molecular volume of the compound. The temperature dependent ΔG_{fus} profile is then approximated by Walden's rule and the heat capacity of fusion estimate $\Delta C_{p,fus} = \Delta S_{fus} = \Delta H_{fus}/T_{melt}$. The predicted solubility of the pigment in a solvent can be calculated as

$$x = \exp\left(\frac{\mu^{pure} - \mu^{solvent} - \Delta G_{fus}}{RT}\right)$$

where μ^{pure} is the chemical potential of the pure pigment and $\mu^{solvent}$ is the chemical potential of the solvent at infinite dilution. To improve the prediction accuracy, we utilized an experimental solubility of 2 mg/L Pigment Yellow 12 in dodecane at 120 °C to calibrate the QSPR results. The ΔG_{fus} values were scaled to match these experimental data as a calibration. Note that due to the lack of solubility data for the decomposed Yellow 12 molecule, the ratio of ΔG_{fus} between Yellow 12 and decomposed Yellow 12 in the QSPR results was maintained. The calibrated ΔG_{fus} values were used to generate the solubility prediction results shown in table S2.

S3 Experimental details of polyethylene (PE) slurry filtration and filter cake deliquoring

The vacuum filtration procedure was adopted from previous report.(*32*) The process was conducted via transferring the PE slurry after cooling and precipitation onto a Buchner funnel. The vacuum was applied by a vacuum pump, as shown in fig. S3A. Paste-like PE was collected for drying after solvent flow stopped.

Mechanical deliquoring was conducted in a custom system shown in fig. S3B. The system comprises the following key items from BVV: a 600X6FJ spool, a HRSJ600 reducer stand, FP600-V3 filter plate, SD1M-6B 1 µm filter, as well as a custom machined aluminum piston. The vessel was filled with ~1.2 L of precipitated polymer slurry above the 1 µm filter, the piston was placed above the slurry, and the vessel was closed and pressurized with nitrogen to drive the piston. Filtration occurred until the solvent flow stopped.

S4 Supplementary data about PE yellowness trend with deliquoring and initial color body concentration

To further support the analysis of color body retention after deliquoring, a series of PE reprecipitation experiments was conducted, with varied initial color body concentration and distinct solvent-to-PE ratio after deliquoring.

It is observed that even after introducing initial concentration as an additional variable, the estimated pigment content in dried PE, according to Eq. (1) in the main text, still has a roughly linear relationship with the PE Yellowness Index. This result supports our hypothesis about pigment behavior and partitioning during the STRAP process.

S5 Solvent cleaning with cold adsorption

In a sustainable dissolution-based plastic recycling process, the solvent separated from the filtration step must be reused in the next batch of polymer dissolution to decrease economic and environmental costs. Before reuse, the solvent should be cleaned to avoid accumulation of dissolved impurities and cross-contamination among different batches. Dissolved color bodies from decomposed diarylide pigments should be removed since they contribute to the yellowness of recycled PE and can potentially perform further side reactions leading to higher light absorptivity.

Distillation and adsorption are common solvent cleaning approaches. Distillation is effective to remove all the non-volatiles from the solvent but is energy intensive.(*31*) Adsorption processes consume less energy and have been applied to remove colorants in plastics recycling processes.(*50*) For example, an adsorption bed is deployed in the PureCycle process of polypropylene purification for color removal.(*50*) With the knowledge of the color sources in solvent and polymers, pertinent investigation is applied in this work for quantitative understanding and targeted optimization of the color removal process.

A makeshift adsorption bed was used to verify the pigment removal from solvents in a continuous system (fig. S5). When using activated carbon (AC) powders (d90 = 90 μm, 90% of the total volume of particle is smaller than 90 μm) to guarantee sufficiently fast adsorption, 150 mL of test solution (Yellow 12 in toluene, 7 mg/L) with weight hour space velocity (WHSV) of 100~150 h^{-1} was turned into colorless solvent by 150 mg adsorbent. After 150 ml, the solvent began to show visible yellow color. The adsorption capacity of the bed was lower than batch experiments but on the same order of magnitude. Applying AC pellets (d~2mm) as an adsorbent resulted in incomplete color removal even with largely reduced WHSV, due to the much lower external surface area and adsorption rate.

As indicated from the adsorption measurements in the main text, AC exhibits improved adsorption capacities and higher rates in dodecanes than toluene, thus an even higher removal efficiency can be expected to counterbalance the reduced concentration gradient in post-STRAP dodecanes. Moreover, the solubility of pigments or decomposed pigments are considerably lower in alkanes, therefore post-STRAP dodecanes contain less color bodies, allowing the adsorption bed to serve longer before replacement.

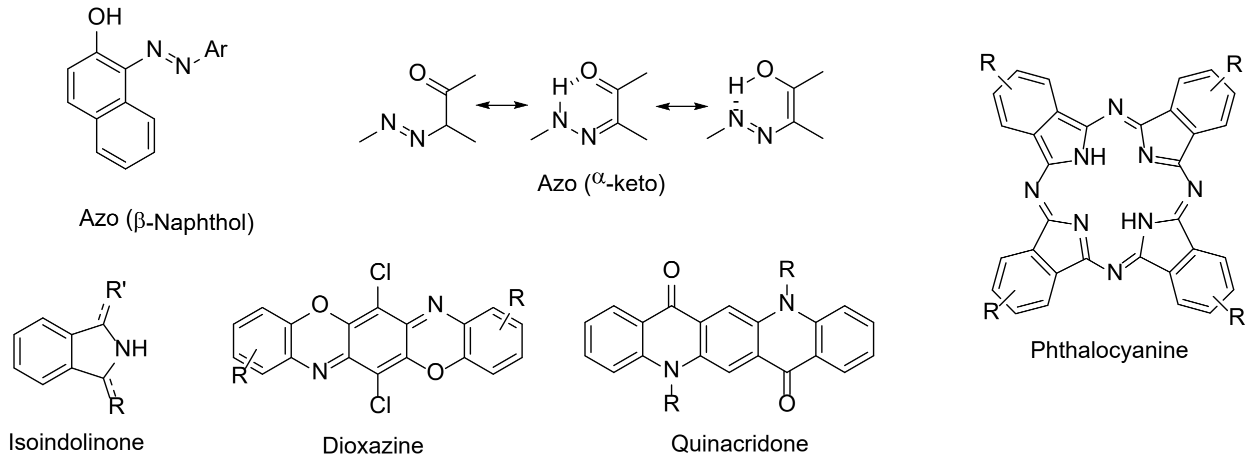

Fig. S1. Representative chromophores in common organic pigments.

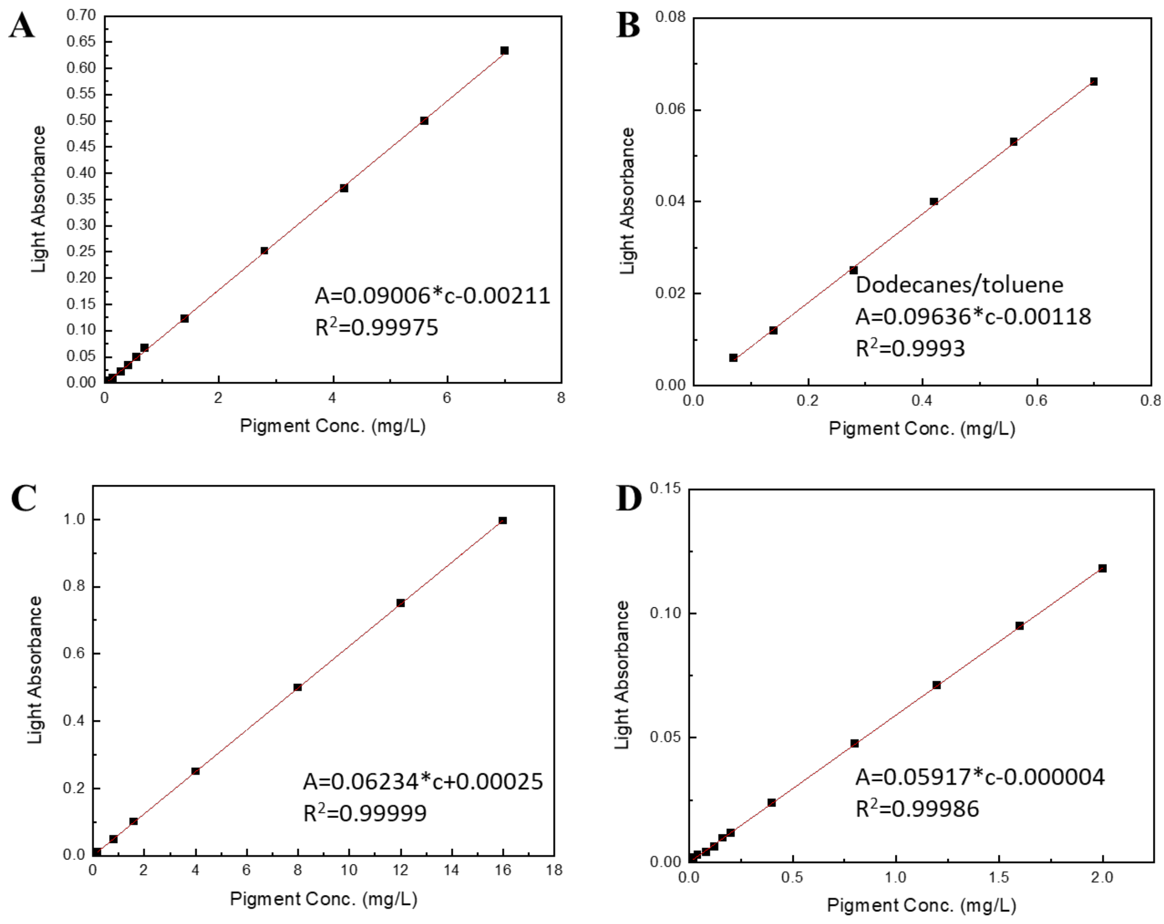

Fig. S2. UV-Vis Calibration of Yellow 12 (A, B) and Yellow 1 (C, D) in toluene (A, C) and toluene/dodecanes (B, D)

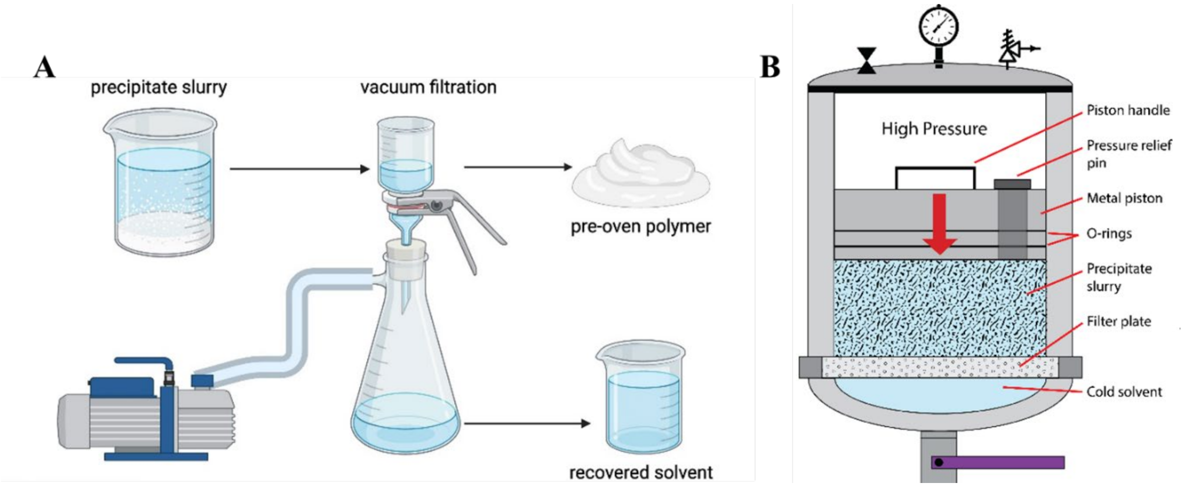

Fig. S3. The cold filtration setup of lab-scale STRAP process. (**A**) Vacuum filtration. (**B**) Compression filtration with a piston.

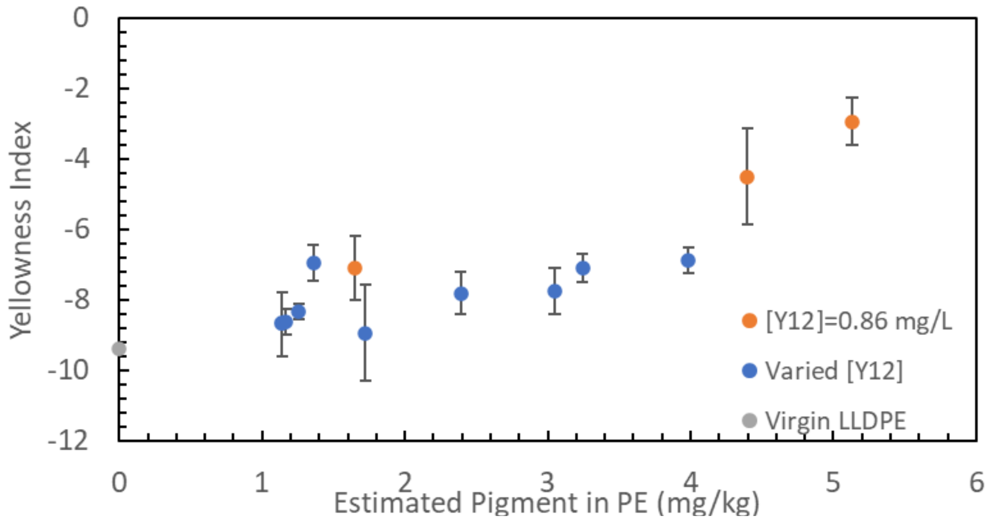

Fig. S4. PE YI vs. estimated pigment content according to Eq. (1). Orange data points are the same as fig. 6B (PE precipitated from initial concentration of 0.86 mg/L decomposed Yellow 12). Blue points represent additional experiments that PE precipitated from a variety of initial decomposed Yellow 12 concentrations.

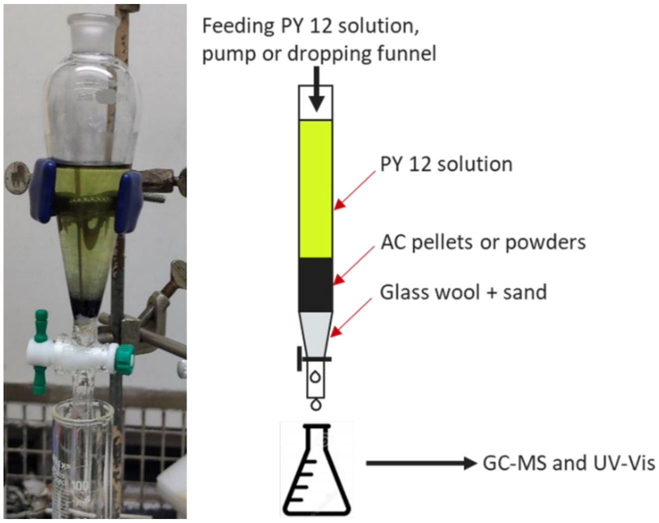

Fig. S5. Simple setup of adsorption bed in the lab to estimate continuous phase adsorption capacity.

Table S1. Common Organic and Inorganic Colorants (adapted from Ref (*6*))

Color	Type	Common Chemical Bases
White	Inorganic	Titanium dioxide, zinc sulfide
Black	Inorganic	Carbon black
Red	Inorganic	Iron oxides
Red	Organic	Azo/diazo, quinacridone
Orange	Organic	Azo/diazo
Yellow	Organic	Azo/diazo
Green	Inorganic	Chromium (III) oxide
Green	Organic	Copper (II) Phthalocyanine
Blue	Inorganic	Cobalt (II) aluminate
Blue	Organic	Copper (II) Phthalocyanine
Violet	Inorganic	Ultramarine violet
Violet	Organic	Quinacridone, dioxazine
Metallic	Inorganic	Aluminum flake, brass
Pearlescent	Inorganic	Coated mica flake

Table S2. COSMO-RS Predicted pigment solubility in different solvents

T (°C)	Pigment solubility (mg/L)											
	Yellow 12				Decomposed Yellow 12				Yellow 1			
	dodecane	heptane	cyclohexane	toluene	dodecane	heptane	cyclohexane	toluene	dodecane	heptane	cyclohexane	toluene
**20**	0.005	0.014	0.028	17.955	0.036	0.080	0.134	14.741	1.706	3.300	5.203	261.325
**30**	0.014	0.038	0.075	34.583	0.099	0.214	0.363	31.010	3.629	6.992	11.095	450.465
**40**	0.035	0.097	0.194	64.963	0.253	0.547	0.935	63.362	7.443	14.283	22.797	761.270
**50**	0.087	0.237	0.477	119.226	0.623	1.338	2.301	126.021	14.760	28.219	45.280	1262.950
**60**	0.205	0.553	1.124	214.128	1.469	3.143	5.435	244.458	28.380	54.077	87.195	2059.290
**70**	0.463	1.243	2.543	376.880	3.339	7.111	12.362	463.320	53.044	100.766	163.208	3303.550
**80**	1.009	2.692	5.545	650.900	7.329	15.548	27.156	859.323	96.593	182.989	297.635	5218.740
**90**	2.125	5.640	11.687	1104.340	15.583	32.936	57.779	1561.850	171.718	324.525	529.980	8124.530
**100**	4.337	11.457	23.872	1842.450	32.169	67.763	119.362	2785.250	298.584	563.148	923.342	12471.900
**110**	8.600	22.618	47.373	3025.190	64.621	135.705	239.970	4878.460	508.691	957.962	1577.120	18885.600
**120**	16.604	43.486	91.523	4891.620	126.564	265.080	470.548	8399.440	850.566	1600.340	2646.350	28213.300
**130**	31.269	81.584	172.491	7792.450	242.147	506.060	901.891	14222.800	1398.110	2630.380	4371.690	41574.400
**140**	57.541	149.612	317.725	12231.200	453.385	946.117	1693.590	23687.500	2262.980	4262.200	7127.320	60394.300
